# Gemin5-dependent RNA association with polysomes enables selective translation of ribosomal and histone mRNAs

**DOI:** 10.1007/s00018-022-04519-4

**Published:** 2022-08-20

**Authors:** Azman Embarc-Buh, Rosario Francisco-Velilla, Juan Antonio Garcia-Martin, Salvador Abellan, Jorge Ramajo, Encarnacion Martinez-Salas

**Affiliations:** 1 Centro de Biología Molecular Severo Ochoa, CSIC-UAM, Nicolás Cabrera 1, 28049 Madrid, Spain; 2 Centro Nacional de Biotecnología, CSIC, Darwin 3, 28049 Madrid, Spain

**Keywords:** RNA-binding proteins, Polysome enrichment, Protein synthesis, TOP mRNAs, Histone mRNAs

## Abstract

Selective translation allows to orchestrate the expression of specific proteins in response to different signals through the concerted action of cis-acting elements and RNA-binding proteins (RBPs). Gemin5 is a ubiquitous RBP involved in snRNP assembly. In addition, Gemin5 regulates translation of different mRNAs through apparently opposite mechanisms of action. Here, we investigated the differential function of Gemin5 in translation by identifying at a genome-wide scale the mRNAs associated with polysomes. Among the mRNAs showing Gemin5-dependent enrichment in polysomal fractions, we identified a selective enhancement of specific transcripts. Comparison of the targets previously identified by CLIP methodologies with the polysome-associated transcripts revealed that only a fraction of the targets was enriched in polysomes. Two different subsets of these mRNAs carry unique cis-acting regulatory elements, the 5’ terminal oligopyrimidine tracts (5’TOP) and the histone stem-loop (hSL) structure at the 3’ end, respectively, encoding ribosomal proteins and histones. RNA-immunoprecipitation (RIP) showed that ribosomal and histone mRNAs coprecipitate with Gemin5. Furthermore, disruption of the TOP motif impaired Gemin5-RNA interaction, and functional analysis showed that Gemin5 stimulates translation of mRNA reporters bearing an intact TOP motif. Likewise, Gemin5 enhanced hSL-dependent mRNA translation. Thus, Gemin5  promotes polysome association of only a subset of its targets, and as a consequence, it favors translation of the ribosomal and the histone mRNAs. Together, the results presented here unveil Gemin5 as a novel translation regulator of mRNA subsets encoding proteins involved in fundamental cellular processes.

**Supplementary Information:**

The online version contains supplementary material available at 10.1007/s00018-022-04519-4.

## Background

Regulation of protein synthesis plays a key role in shaping the cellular proteome. Indeed, regulation of translation is a prominent mechanism among post-transcriptional pathways affecting the abundance of proteins [1]. Such mechanisms can be general, affecting most transcripts in the cell, or selective, by regulating a subset of related mRNAs. Selective translation is essential for the cellular response to physiological and stress conditions, which in turn ensures adaptation and survival of all organisms [2].

Gemin5 is a predominantly cytoplasmic RNA-binding protein (RBP) that recognizes and delivers the small nuclear RNAs (snRNAs) to the survival of motor neurons (SMN) complex [3, 4]. This process allows the assembly of the small ribonucleoproteins (snRNPs), which are essential components of the spliceosome [5]. However, a large part of Gemin5 is found outside of the SMN complex [6], suggesting that it may perform additional functions. Consistent with this notion, this protein participates in various cellular processes, including tissue regeneration [7], recognition of 7S RNA Signal Recognition Particle (SRP) [8], trans-splicing [9], and translation control [10]. In further support of the critical activities of this ubiquitously expressed protein for cell growth, recent studies showed that human *Gemin5* variants cause developmental disorders presumably due to decreased levels of endogenous Gemin5 protein [11–14], summing up to the observation that a null KO mouse is embryonic lethal [12], as well as in zebrafish and Drosophila [13, 15].

The multifunctional activities of Gemin5 rely on its modular organization, consisting of different domains that enable the association of a wide range of RNAs and proteins. Gemin5 contains at the N-terminal half a 14 WD repeat domain that recognizes the Sm-site of snRNAs and the m^7^G cap via base-specific interactions [16–18]. The middle region comprises a TPR-like domain responsible for Gemin5 dimerization [19]. In addition, Gemin5 harbors at the most C-terminal part a non-canonical RNA-binding site [20].

Regarding its role in translation control, Gemin5 was identified as a ribosome-binding protein and as a negative regulator of protein synthesis [21–23]. However, recent studies have shown that Gemin5 can stimulate translation of selective mRNAs. Gemin5 interacts with a structural element present on its own mRNA stimulating translation, providing a positive feedback loop that auto-regulates its cellular levels [10]. In another study, it was found that Gemin5 binds to the 3’UTR of the SMN mRNA enhancing its translation [24]. Moreover, Gemin5 was identified as a factor bound to viral internal ribosomal entry site (IRES) elements down-regulating IRES-dependent translation [23]. Gemin5 also interacts with the viral RNA of Sindbis virus [25]. Collectively, these results suggest that Gemin5 plays a key role in selective translation recognizing structural elements of its RNA targets.

The dynamic interplay between RBPs and mRNA cis-acting elements regulates translation using distinct mechanisms of action [26–28]. The still underestimated functions of cis-acting regulatory elements, in many cases combining sequence and RNA structure, is exemplified by IRES elements, AU-rich elements (AREs), 5’terminal oligopyrimidine tracts (5’TOP), or the metazoan histone stem-loop (hSL) structures, among others. The 5’TOP motif is typically present in mRNAs that encode proteins related to translation, including all ribosomal proteins [29]. Translation of the TOP mRNA family is mediated by trans-regulators that recognize this motif in response to different physiological conditions [30–32]. Another special case of translation is represented by the histone mRNA family, a unique group of not polyadenylated mammalian mRNAs [33, 34]. The mature histone mRNAs are generated by a single endonucleolytic cleavage between two cis-acting elements. Upstream of the cleavage site there is a conserved hairpin structure, designated hSL, recognized by the stem-loop-binding protein (SLBP), while the downstream element anneals to the 5’end of the minor U7 snRNP [35].

Here, we sought to identify at a genome-wide scale mRNAs translationally regulated by Gemin5. We show that Gemin5 up-regulates processes related with protein synthesis and nucleic acid metabolism. Among these transcripts, we have found nearly all mRNAs encoding ribosomal proteins and several histone mRNAs, which bear specific cis-regulatory elements. RNA immunoprecipitation studies revealed the binding of Gemin5 with these mRNAs in the cellular context. Functional translation assays confirmed that Gemin5 regulates translation of TOP and histone mRNA reporters. Together, our results shed light on the mRNAs selectively regulated by Gemin5 at the translational level by the specific recognition of RNA-regulatory elements.

## Results

### Gemin5 modulates the association with polysomes of different subsets of mRNAs

**Fig. 1:**
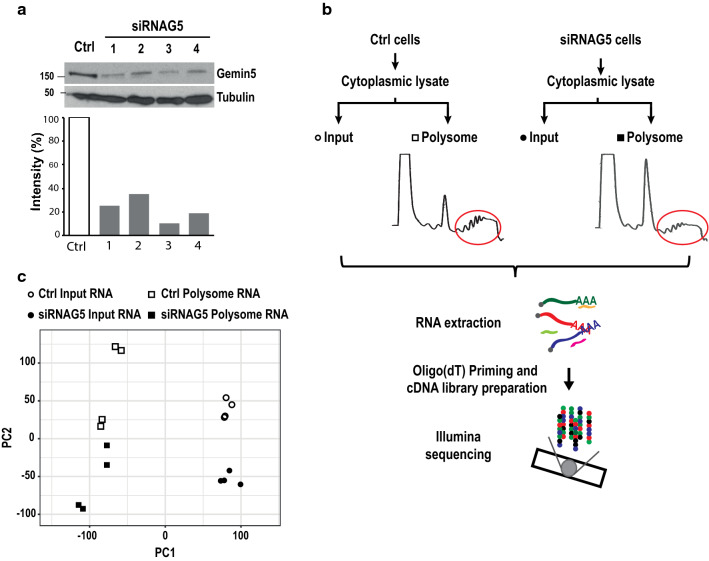
Flow-chart of the approach used to analyze Gemin5-dependent regulation of polysome associated RNAs. **a** Immunoblotting of Gemin5 prepared from control (Ctrl) and 4 independent samples of Gemin5-depleted cells (siRNAG5) using anti-Gemin5 antibody. Tubulin was used as loading control. The quantification of the Gemin5 silencing in four independent replicas relative to siRNA control (Ctrl) is shown below. **b** Overview of the procedure carried out to identify mRNAs associated with polysomes in Gemin5-depleted cells. Cytoplasmic lysates from 4 independent biological replicates of control (Ctrl) and Gemin5-depleted cells (siRNAG5) were prepared. RNA was extracted from Input samples and from the combined polysomal fractions (depicted by a red oval). After oligo(dT)-primed cDNA libraries preparation, mRNAs were identified by Illumina sequencing. **c** Principal component analysis (PCA) of Input and Polysome samples prepared from Ctrl and siRNAG5 cells

To identify the mRNAs associated with translationally active polysomes regulated by Gemin5, we prepared four independent biological samples of Gemin5-depleted HEK293 cells (siRNAG5) and control cells (Ctrl). Gemin5 depletion, ranging between 10 and 35%, was verified by western blot (Fig. 1a). Then, individual cell lysates were subjected to polysomal fractionation. In each case, the mRNAs present in polysomes (Polysome) and cytoplasmic cell lysates (Input) were extracted and analyzed by RNA-Seq (Fig. 1b). Principal component analysis (PCA) showed minimal variations among the four biological replicates of each condition (Fig. 1c), confirming the reproducibility of the assay.

**Fig. 2:**
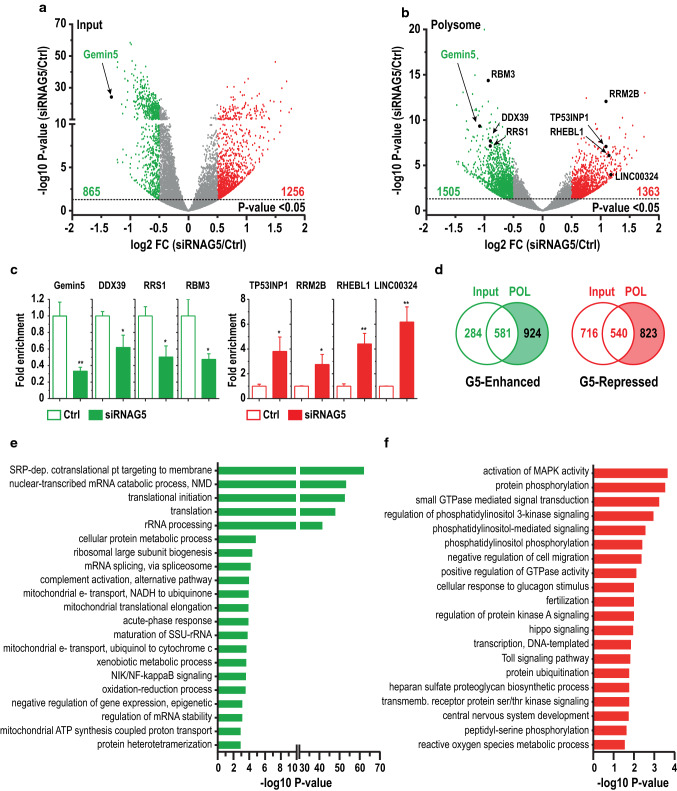
Identification of mRNAs differentially associated with polysomes in Gemin5-depleted cells. Volcano plots showing the log2 FC (X) versus − log10 *P* value (Y) of each mRNA detected in the RNA-Seq analysis from the four replicas for the Input (**a**), and the Polysome (**b**), using as cutoff − 0.5 > log2 FC > 0.5 and *P* < 0.05. A black arrow depicts the detection of Gemin5 RNA in the Input and the Polysome. **c** RTqPCR validation of polysome-bound mRNAs using specific primers for Gemin5, DDX39, RRS1, RBM3, TP53INP1, RRM2B, RHEBL1, and LINC00324. **d** Venn diagrams depicting the overlapping mRNAs significantly enhanced or repressed by Gemin5 in the Input and Polysome (POL) samples, considering the mRNAs not significantly altered in the Input samples. Gene ontology classification obtained for mRNAs belonging to G5-Enhanced (**e**) and G5-Repressed groups (**f**). The top GO terms of the biological processes are represented according to *P* value; cutoff was set to 10^–3^

The statistical analysis of the mRNAs differentially altered in the mRNA pool (Input) in Gemin5-depleted cells compared to control cells (*P* < 0.05 and log2 FC < − 0.5 or log2 FC > 0.5) showed 865 genes with significantly reduced read counts, while these were significantly enriched for 1256 genes (Fig. 2a), indicating that Gemin5 regulates the steady state of cellular mRNAs. We noticed a large number of downrepresented transcripts with high -log10 *P*-value compared to the overrepresented RNAs. Detailed analysis of the down-represented group of transcripts identified networks related to RNA splicing, Ribosome biogenesis, tRNA modification, Translation, and Mitochondrial transport (Fig. S1). None of these networks were detected within the overrepresented mRNAs that instead contained an Antigen processing and presentation network.

For the polysome-bound mRNAs (Polysome), we identified 1505 genes with significantly reduced read counts, whereas a significant enrichment was observed for 1363 genes (Fig. 2b). Thus, the number of mRNAs differentially associated with polysomes (2868) was 26% greater than those showing altered levels in total lysates (2121), suggesting that Gemin5 exerts a higher impact on translation than in the steady state of mRNAs. As expected from the silencing strategy, the Gemin5 mRNA was significantly decreased in siRNAG5 cells in both cases, the input and the polysome (Fig. 2a and b). RTqPCR validation of Gemin5 mRNA and seven randomly selected mRNAs, including down-represented (DDX39, RRS1, and RBM3) and overrepresented genes (TP53INP1, RRM2B, RHEBL1, and LINC00324) in polysome Gemin5-depleted relative to control cells, fully confirmed the reliability of the RNA-Seq data (Fig. 2c).

Considering the functional relevance of Gemin5 for the assembly of the SMN complex [5], as well as its role in selective translation [10, 24], we examined the effect of Gemin5 silencing on the mRNA abundance of members of the SMN complex. Concomitant reduced levels of Gemin5 and Gemin6 mRNAs were observed. However, the remaining SMN complex mRNAs (Gemin2, 4, 8, and Smn1-2) were below the cutoff criteria (Fig. S2). Furthermore, we observed Gemin6 and Gemin7, but not Smn1-2, within the group of mRNAs significantly enriched in polysomes.

To investigate the mRNAs whose translation could be selectively regulated by Gemin5, we selected mRNAs showing significant changes in polysomes but remained constant in the Input samples. These criteria were imposed to avoid post-transcriptional effects that might influence their association to polysomes. Using these cutoff criteria, we identified 924 mRNAs downrepresented upon depletion of Gemin5 (henceforth termed as G5-Enhanced), and 823 genes overrepresented in siRNAG5 cells (from now on termed G5-Repressed) (Fig. 2d and Dataset 1). These results suggest that Gemin5 can act as a down-regulator of protein synthesis for a subset of mRNAs, but also as a translation stimulator for a different group of mRNAs.

### Gemin5 enhances polysome association of functionally related mRNA subsets

The biological pathways regulated by Gemin5 were analyzed using DAVID V6.8 [36]. The GO terms obtained with G5-Enhanced mRNAs were greatly enriched (*P* values 10^–63^–10^–42^) in various steps related to protein synthesis (Fig. 2e), such as SRP-dependent cotranslational protein targeting to membrane, nuclear-transcribed mRNA catabolic process, nonsense-mediated decay, translational initiation, translation, and rRNA processing. Other processes identified in this group (*P* values 10^–5^–10^–4^) were the ribosomal large subunit biogenesis, mRNA splicing via spliceosome, mitochondrial translation elongation, maturation of SSU-rRNA, or regulation of mRNA stability (Fig. 2e). Conversely, the GO terms identified in the G5-Repressed group display much lower *P* values (10^–4^–10^–3^) including activation of MAPK activity, protein phosphorylation, small GTPase mediated signal transduction, and regulation of phosphatidylinositol 3-kinase signaling (Fig. 2f). Of note, the top G5-Enhanced processes have about 20-fold higher statistical significance than the G5-Repressed ones, despite the number of transcripts significantly altered is similar in both groups (924 and 823, respectively) (Fig. 2d). These results suggest that G5-Enhanced mRNAs encode for proteins belonging to closely related pathways, whereas G5-Repressed transcripts encode proteins with diverse, separate functions.

**Fig. 3:**
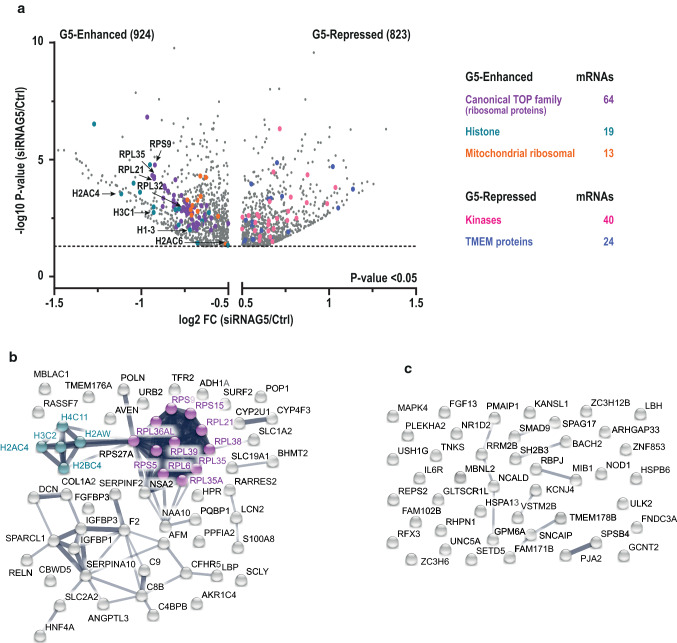
G5-Enhanced mRNAs encode for functionally related proteins. **a** Volcano plots showing mRNAs exclusively altered in polysomes (G5 Enhanced and G5 Repressed). The position of individual representative enhanced mRNAs (marked by color) is indicated. STRING networks of the proteins encoded by the most significantly altered mRNAs from the polysome-bound mRNAs in G5-Enhanced (**b**), or in G5-Repressed (**c**) groups using as a cutoff criteria − 0.8 > log2 FC > 0.8 and *P* < 5 × 10^–4^

The mRNAs differentially associated with polysomes revealed the presence of several groups encoding protein families (Fig. 3a). Among the G5-Enhanced mRNAs we observed 64 of the 97 mRNAs containing canonical 5’terminal oligopyrimidine tracts (5’TOP) (Table S1), which encode mostly translation factors and ribosomal proteins [29]. Translation of these transcripts is controlled by the 5’TOP motif. Moreover, Gemin5 depletion reduces the association to polysomes of 19 mRNAs encoding histones, 8 of them belonging to the H2A family (Fig. 3a and Table S2). These transcripts contain a conserved stem-loop (hSL) at the 3’ end of the mRNA that regulates their processing and translation [34]. The high number of mRNAs identified carrying 5’TOP or hSL motifs suggested that Gemin5 could regulate their translation by binding to these cis-regulatory elements.

Additionally, the G5-Enhanced group contains 13 mRNAs encoding mitochondrial ribosomal proteins, 5 Sm proteins (small nuclear ribonucleoprotein B, D1, D2, D3 and E), which are the core components of the U1, U2, U4 and U5 snRNPs [5], and 4 Sm-like proteins (LSM1, 4, 6 and 7) (Dataset 1). Other mRNAs present in this group encode members of the serpin family, proteins of cytochrome P450, and factors belonging to proteasome subunit (Dataset 1). Conversely, within the G5-Repressed group (Fig. 3a) we identified 40 transcripts that encode kinases involved in different pathways, and 24 mRNAs encoding transmembrane (TMEM) proteins predicted as components of cell membranes [37].

Next, we focused our attention on the mRNAs showing the highest differences between the fold-change value in Gemin5-depleted cells relative to control ones in polysomes (*P* < 5 × 10^–4^ and log2 FC < − 0.8 or log2 FC > 0.8). The most significantly altered transcripts in G5-Enhanced mRNAs encode ribosomal proteins, histones, serpins, and cytochrome P450 family factors (Table S3). To define the network(s) of proteins encoded by these mRNAs we used STRING [38]. This analysis revealed that the closest relationships take place in ribosomal and histone mRNAs (Fig. 3b). In contrast, the G5-Repressed group (Fig. 3c and Table S4) contains transcripts encoding functionally unrelated factors.

In conclusion, the Gemin5-Enhanced mRNAs showing the closest relationship among them encode ribosomal and histone proteins. Given that each of these mRNA families have characteristic unique cis-regulatory elements, the 5’TOP motif or the hSL hairpin, respectively, we suggest that Gemin5 may favor differential translation of these mRNAs likely through interaction with these cis-acting elements.

### Gemin5 favors translation of a subset of its targets

**Fig. 4:**
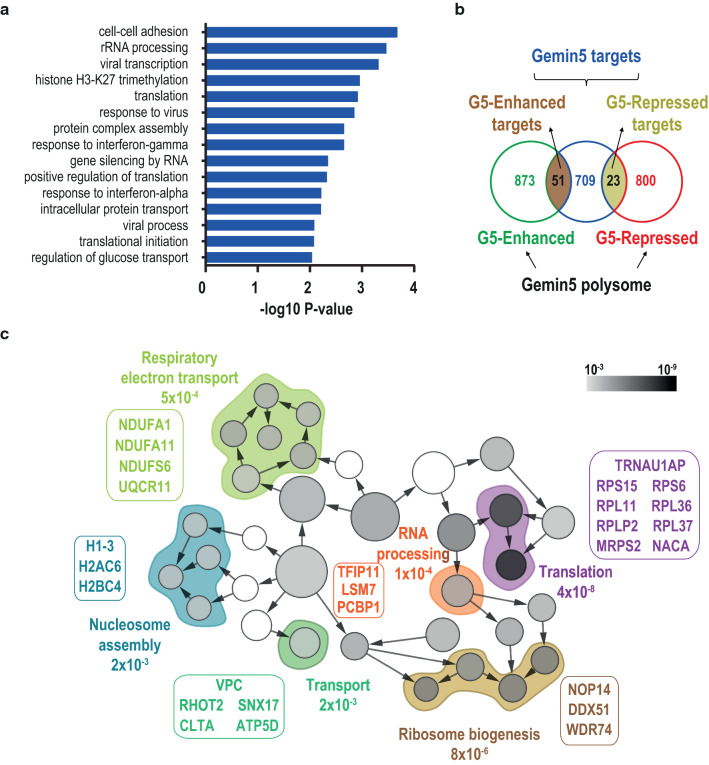
G5-Enhanced targets encode for ribosomal proteins and histones. **a** Gene ontology classification obtained for mRNAs previously identified as Gemin5 cellular targets. The top GO terms of the biological processes are represented according to *P* value. **b** Venn diagrams depicting the overlapping mRNAs between Gemin5 targets and G5-Enhaced or G5-Repressed groups. **c** Functional networks obtained with BiNGO (Cytoscape platform) of proteins encoded by mRNAs belonging to G5-Enhanced targets group. The *P* values of the networks relative to a complete human proteome, and the proteins that compose the nodes included in each network are indicated

Recent cross-linking immunoprecipitation (CLIP)-based studies reported genome-wide mRNA targets of Gemin5 [10, 39] (https://www.encodeproject.org/experiments/ENCSR238CLX/). Among others, the GO terms obtained with the mature mRNA targets (783) showed that Gemin5 ligands are involved in cell–cell adhesion, rRNA processing, histone metabolism, and translation (Fig. 4a). Some of these terms coincide with the processes significantly increased in the polysome-bound mRNAs upon depletion of Gemin5 (Fig. 2e). These results suggest that only a fraction of the Gemin5 targets is differentially engaged in polysomes.

To determine the relationship between these two sets of data, Gemin5 targets defined by CLIP and Gemin5-driven association to polysomes, we analyzed the overlap between mRNAs altered in polysomes upon depletion of Gemin5 and the mRNAs described as Gemin5 targets. This analysis shows that Gemin5 stimulates polysome engagement of 51 mRNAs out of the 783 Gemin5 interactors (G5-Enhanced targets), while it decreases the polysome engagement of 23 mRNAs (G5-Repressed targets) (Fig. 4b). We analyzed the GO biological processes of the proteins encoded by the mRNAs included in the G5-Enhanced targets and G5-Repressed targets groups using BiNGO (Cytoscape platform). This study showed a distribution in statistically significant overrepresented nodes, grouped in functional networks according to the biological process. Only in the case of G5-Enhanced targets group, we obtained significant nodes. The top functional networks are Translation (*P*-value 8 × 10^–8^) and Ribosome biogenesis (*P*-value 8 × 10^–6^), consistent with the top G5-Enhanced terms (rRNA processing, translation, ribosomal subunit biogenesis) (Figs. 3a and 4c). Other functional networks identified in this group (*P* values 10^–4^–10^–3^) were Respiratory electron transport, Nucleosome assembly, Transport, and RNA processing.

Of note, the functional interaction network encoded by G5-Enhanced targets highlights again the enrichment of 5’TOP and histone mRNAs (Figs. 4c and S3). The systematic analysis of the Gemin5 mRNA targets shows that this protein interacts with 10 and 11 5’TOP and histone mature mRNAs, respectively (Table S5). These results led us to hypothesize that Gemin5 selectively enhances the translation of these transcripts via Gemin5-mRNA interactions.

### Gemin5 positively regulates 5’TOP-dependent translation

**Fig. 5:**
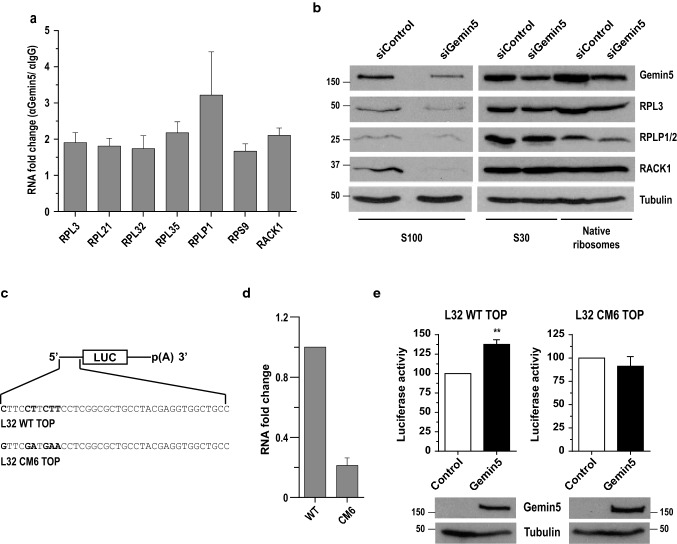
Gemin5 stimulates the translation of TOP mRNAs via Gemin5-TOP motif interaction. **a** RNA immunoprecipitation (RIP) assays of cell lysates using anti-Gemin5 or anti-IgG to immunoprecipitate RNA–protein complexes. RNA was isolated and retrotranscribed to cDNA for qPCR analysis of the indicated TOP mRNAs. **b** Cellular fractions corresponding to S100, S30 and native ribosomes were obtained from control and Gemin5-depleted cells. The fractions were analyzed by western blot to detect Gemin5, RPL3, RPLP1/2, and RACK1. Tubulin was used as a loading control. **c** Diagrams depicting the constructs harboring L32 WT TOP or the mutant L32 CM6 TOP motif used in the affinity purification and translation assays. **d** RNA fold change observed in the His-Gemin5 protein purification analysis from lysates expressing the constructs pL32-WT-luc or pL32-CM6-luc. **e** Effect of the overexpression of Gemin5 on 5’TOP-dependent translation measured by luciferase activity using the reporter constructs described in C, relative to the values observed in control cells transfected with the empty vector. The luciferase activity values (RLU/µg protein) obtained in one representative experiment performed side by side for pL32-WT-luc and pL32-CM6-luc were 487,613 and 292,815, respectively. Expression of Xpress-Gemin5 was detected with anti-Xpress and tubulin was used as loading control. Each experiment was repeated at least three times. Values represent the mean ± SEM. (**P < 0.01 by Student’s *t* test)

The results mentioned above prompted us to investigate the role of Gemin5 as a regulator of 5’TOP-dependent translation. To validate the existence of complexes comprising Gemin5 and the TOP mRNAs, we performed RNA immunoprecipitation (RIP) assays. The fold change of seven different TOP mRNAs (RPL3, RPL21, RPL32, RPL35, RPLP1, RPS9, and RACK1) was tested by RTqPCR in the immunoprecipitated Gemin5 complexes relative to a control IgG. These mRNAs were significantly enriched (~ twofold) in αGemin5 RIP samples compared with control IgG RIP (Fig. 5a), showing the interaction of Gemin5 with the ribosomal mRNAs.

Next, we determined if Gemin5 silencing altered the abundance of ribosomal proteins. Since ribosomal proteins are very stable following ribosome assembly, we studied the effect of Gemin5 depletion on the level of ribosomal proteins that are immunodetectable in cytoplasmic lysates free of ribosomal particles (S100 fraction) (Fig. 5b). Ultracentrifugation of the total cytoplasmic lysates (S30 fraction) renders soluble proteins (S100 fraction, supernatant) and native ribosome particles (pellet). Interestingly, Gemin5 silencing reduced the levels of the proteins RPL3, RPLP1/2 and RACK1 in S100 fractions compared to the control siRNA treatment (Fig. 5b). These results indicate a positive relationship between the cytoplasmic levels of free ribosomal proteins with the capacity of Gemin5 to alter the association of ribosomal mRNAs with polysomes.

The 5’UTR of TOP mRNAs is characterized by the presence of a cis-regulatory element that consists of a cytosine residue at the 5’, followed by a stretch of 5–15 pyrimidines and a CG-rich region immediately downstream of the motif [29, 40]. To determine whether the Gemin5 interaction with ribosomal mRNAs depends upon the TOP motif, we employed the RPL32 5’UTR construct which retains such characteristics [29]. The pL32-WT-Luc construct contains 189 nt from the RPL32 gene encompassing the transcriptional start site and 38 nt of exon 1 including the 5’TOP motif upstream of firefly luciferase coding region (Fig. 5c). In addition, to examine the interplay between the TOP motif and Gemin5, we substituted six pyrimidines within the 5’TOP motif by purines, creating pL32-CM6-Luc (Fig. 5c). Similar mutations have been shown to abolish 5’TOP properties [41]. Each of these constructs were transfected in HEK293 cells with a plasmid expressing His-Gemin5 prior to perform pull-down assays to analyze the interaction of Gemin5 with these transcripts (Fig. 5d). The comparison of the RNA-fold change observed after RTqPCR analysis of the RNA found within the His-Gemin5 complexes indicated that Gemin5 interacts ~ fivefold more with RNA carrying the L32-WT TOP sequence than the mutant L32-CM6 TOP.

Then, to study the interdependence between Gemin5-TOP motif interaction and translation regulation we measured the effect of Gemin5 expression on L32WT-TOP-luc and mutant L32CM6-TOP-luc protein synthesis (Fig. 5e). Overexpression of Gemin5 in HEK293 cells enhanced the luciferase activity expressed from the pL32-WT-Luc, but not from the pL32-CM6-Luc, relative to control cells. In contrast, depletion of Gemin5 by siRNA resulted in a statistically significant decrease of luciferase activity from the L32 WT TOP mRNA, but not of the mutant L32 CM6 TOP, relative to a control siRNA (Fig. S4). Therefore, we conclude that Gemin5 behaves as a translation stimulator of TOP-containing mRNAs in intact TOP-dependent manner.

All together, these data allow us to conclude that Gemin5 enhances the translation of ribosomal mRNAs in the cellular context via direct or undirect Gemin5-TOP interactions. The mechanism is yet to be elucidated, however, it is probably related with the formation of RNP complexes.

### Gemin5 stimulates the translation of histone mRNAs

**Fig. 6:**
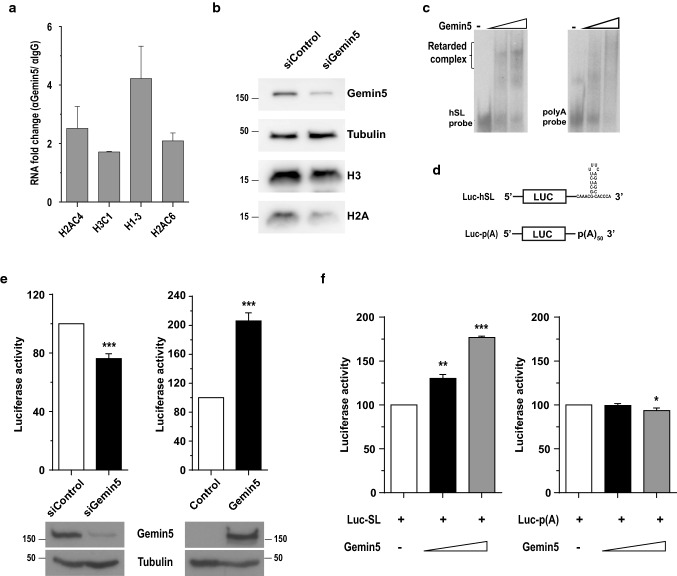
Gemin5 stimulates the translation of histone mRNAs through Gemin5-hSL binding. **a** RIP analysis of cell lysates using anti-Gemin5 or anti-IgG to immunoprecipitate RNA–protein complexes. The isolated RNA was subjected to RTqPCR analysis of the histone mRNAs indicated. **b** Whole cell extracts were obtained from cells treated with control or Gemin5 siRNAs and were analyzed by western blot to immunodetect Gemin5, H3, and H2A. Tubulin was used as loading control. **c** Gel-shift assays conducted with purified Gemin5 protein and either radiolabeled folded histone hSL RNA, or polyA probe. **d** Diagram representing the reporter constructs used in the translation assays which consist in the ORF of the firefly luciferase followed by the histone stem-loop (Luc-hSL) or a poly-A tail, Luc-p(A), at the 3’UTR. **e** Effect of the Gemin5 silencing and Gemin5 overexpression on the translation of the histone mRNA reporter Luc-SL. Gemin5-depleted and control cells (left), or cells overexpressing Gemin5 and control cells transfected with the empty vector (right) were transfected with the reporter Luc-SL to monitor luciferase activity. Gemin5 silencing was monitored by western blot using anti-Gemin5 antibody, while expression of Xpress-Gemin5 was detected with anti-Xpress. Tubulin was used as loading control in both cases. **f** In vitro translation assays using Gemin5 purified protein with the reporter construct Luc-hSL, and the transcript Luc-p(A) as a control. Each experiment was repeated at least three times. Values represent the mean ± SEM (***P < 0.005; **P < 0.01; *P < 0.05 by Student’s *t* test)

The silencing of Gemin5 resulted in a decrease of the association to polysomes of 19 histone mRNAs (Fig. 3a), the second largest group of altered mRNAs. Likewise, we found histone mRNAs within the G5-Enhanced transcripts (Fig. 3b and Table S2). To reinforce the idea that Gemin5 forms part of RNP complexes containing histone mRNAs, we performed RIP assays. The RTqPCR analysis of affinity-purified Gemin5 complexes using specific primers for distinct histone mRNAs showed significant enrichment of H2AB, H3A, H1D and H2AC (~ 1.8–fourfold) relative to control IgG RIP samples (Fig. 6a). This encouraged us to study the role of Gemin5 in histone mRNA translation. We observed that the abundance of H2A and H3A proteins in total protein content was reduced upon Gemin5 depletion (Fig. 6b).

These findings and the presence of the consensus stem-loop (hSL) in the 3’UTR of histone mRNAs [42], prompted us to ask if Gemin5 interaction with histone mRNAs is mediated by this cis-regulatory element. To test this hypothesis, we incubated purified Gemin5 protein with a folded uniformly labelled hSL RNA to assemble RNP complexes. Detection of a retarded complex in native gels in the presence of Gemin5 protein indicates the formation of Gemin5-hSL complex (Fig. 6c). In contrast, no similar band shift was observed when Gemin5 was incubated with a polyA probe. Since the histone 3’ terminal stem-loop structure regulates the translation of these mRNAs we investigated if Gemin5 might regulate their translation through this cis-regulatory element. For this, we used a linearized Luc-hSL reporter construct harboring the histone stem-loop at the 3’end of the Luciferase coding region (Fig. 6d). Luciferase activity was reduced in Gemin5-depleted cells relative to control ones (Fig. 6e), suggesting that Gemin5 modulates hSL-dependent translation. Next, the reciprocal experiment, increasing the cellular levels of Gemin5 protein showed about twofold translation stimulation of Luc-hSL relative to control cells (Fig. 6e). Overall, these results suggest that Gemin5 modulates hSL-dependent translation.

Additionally, in vitro translation assays conducted in the presence of Gemin5 protein with Luc-hSL RNA and Luc-p(A), a construct carrying 50-nt poly(A) tail instead of hSL (Fig. 6d), showed that Gemin5 stimulates the translation of the RNA reporter containing the histone 3’-terminal stem-loop structure, but not of the Luc-poly(A) reporter, in a dose-dependent manner (Fig. 6f). These results confirm that the translation stimulatory effect of Gemin5 requires the hSL sequence. Collectively, our results indicate that Gemin5 enhances histone mRNA translation via binding to the 3’ terminal stem-loop structure.

## Discussion

The function of RNA-binding proteins is tightly connected to their RNA targets, such that their expression level may differentially affect distinct processes. Here, we show that the multifaceted protein Gemin5 is responsible for the preferential partitioning of mRNAs into polysomes. Using a siRNA depletion strategy, we have observed that reduced levels of expression of Gemin5 cause a switch on the relative abundance, both decreasing and increasing, of nearly 3000 mRNAs associated with polysomes. This result indicates that the protein can perform opposite functions depending on the RNA subset.

The study of the underrepresented mRNAs in polysomes in Gemin5 silenced cells, thereby showing an enhanced effect in translation (G5-Enhanced), revealed that a significant number of factors encoded by these mRNAs participate in cellular processes related with translation and RNA metabolism (Fig. 2e). In contrast, the cellular processes overrepresented in the G5-Repressed mRNAs show lower significance values according to GO (Fig. 2f), and besides, are mostly linked to signaling processes (Fig. 3c). Moreover, the detailed analysis of the translationally stimulated G5-Enhanced mRNAs showed the presence of mRNAs encoding specific protein families among a large number of general RNA-binding proteins (Fig. 3b). Notably, the groups of mRNAs with the highest number of components are the ribosomal protein and the histone (Fig. 3a). Since both mRNA families bear unique cis-regulatory elements within their sequences, we argued that Gemin5 could regulate translation of this specific mRNAs via RNA–protein interactions.

The presence of specific RNA motifs within mRNAs is thought to be a signature for the interaction with a given protein, which in turn, will determine the fate of the RNA target. Previous data have shown that the Gemin5 protein interacts with a variety of RNAs. One of the best characterized are snRNAs, which contain a U-heptad near a hairpin that constitutes the recognition motif for Gemin5 [3, 43]. Another motif present in the RNA targets of Gemin5 is the m^7^GTP (cap), shared by mRNAs and snRNAs [17]. Furthermore, the unbiased identification of Gemin5 targets in K562 human cells revealed an enrichment of mRNA regions in the 5’UTR and peaking at the start codon [39]. Another study using the non-canonical RNA-binding site of Gemin5 identified mRNA targets in HEK293 human cells enriched in G:C content, thereby prone to adopt relatively stable secondary structure [10]. This feature is shared with the IRES element and an internal regulatory region of the Gemin5 mRNA coding sequence [45]. In these cases, it has been reported that the direct interaction of the RNA with Gemin5 determines the translation efficiency of the corresponding mRNA [44]. Collectively, these data suggest that RNA conformation is a determinant factor for Gemin5 association.

**Fig. 7:**
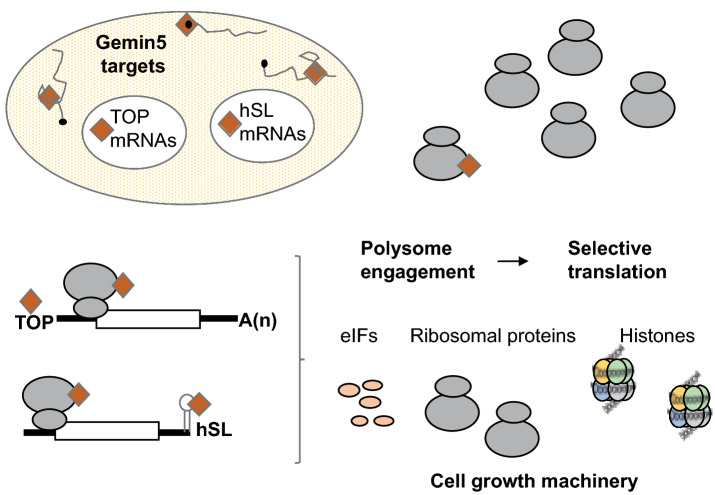
Proposed model for the role of Gemin5 on selective translation. Gemin5 interacts with various mRNA targets in a sequence and structure-dependent manner. Only a fraction of Gemin5 mRNA targets are selectively associated with polysomes. Gemin5-tagged ribosomes are preferentially involved in translation of a subset of Gemin5 targets, such as TOP and hSL mRNAs, encoding pivotal components of the cell growth machinery. Gemin5 is depicted as a diamond, a black circle denotes the cap of mRNAs. The empty ovals represent TOP (ribosome and eIFs) and hSL (histone mRNAs). We hypothesize that Gemin5 silencing concurs with reduced synthesis of ribosomal proteins and histones, among other cellular factors, resulting in significant reduction of cell components required for continuous cell growth, in accordance with the notion that loss of Gemin5 function is detrimental for cell survival

Comparison of the previously identified Gemin5 targets using CLIP-based methodologies [10, 39], with the transcripts identified in the current study revealed that only a fraction of the mRNA targets is shared by both set of data (Fig. 4b). This result highlights two separate features of Gemin5, namely the ability to interact with RNA and the function relying in differential polysome association, finally allowing selective translation (Fig. 7). Given the multitasking activity of this protein, we anticipate different mechanisms towards translation regulation presumably guided by protein interactions and/or still unknown ligands. Thus, analysis of the mechanism of action mediating translation of additional mRNAs within the group of G5-Enhanced mRNAs will need future studies. Considering the salient properties of the known targets of Gemin5, we focused our attention in two groups, the TOP mRNAs and the histone mRNAs. TOP mRNAs generally encode ribosomal proteins and translation factors [29]. These transcripts are characterized by a distinctive motif at the 5’end, that contains a cytosine after the m^7^GTP structure followed by a long uninterrupted pyrimidine tract [41]. In contrast to the vast majority of eukaryotic mRNAs, the mammalian histone mRNAs are not polyadenylated at their 3’end and instead, the mRNA ends in a conserved stem-loop, termed hSL [42].

Using RNA-immunoprecipitation, we found a significant increase in the amounts of mRNA associated with Gemin5 encoding various ribosomal proteins, all of which contain the TOP motif at their 5’end. We also showed that mutations in the TOP motif abrogate Gemin5 interaction, as indicated by the fivefold decrease in RNA fold change compared to the TOP WT. Moreover, we observed an increase in luciferase expression in cells overexpressing Gemin5 relative to control cells when we used reporters carrying the TOP WT, which was not observed in the reporter carrying the TOP mutant. Consistent with these data, reduced levels of Gemin5 are accompanied by a reduced level of ribosomal proteins (Fig. 5b). These results reinforce the idea that Gemin5 not only has the capability to interact with TOP mRNAs, but also influences the efficiency of translation. Thereby, Gemin5 regulates the synthesis of ribosomal proteins, and likely other mRNAs harboring TOP motifs. All these factors belong to the translational machinery and have pivotal roles in shaping the cellular proteome.

Another group of transcripts identified in our study within the G5-Enhanced targets encode histones. We showed that Gemin5 silencing resulted in a decreased translation of reporters carrying the hSL hairpin while its overexpression enhanced translation of the hSL reporter. Conversely, Gemin5 stimulates in vitro translation of reporters carrying this hairpin in comparison to conventional polyadenylated reporters. In addition, a transcript carrying the consensus stem-loop of histones forms a retarded complex with Gemin5 protein, supporting the hypothesis that interaction of Gemin5 with the 3’UTR of histone mRNAs is at the basis of histone mRNA translation regulation.

Recent studies have shown that loss of function of Gemin5 is detrimental for cell survival, and mutant forms of the protein showing failures in RNA-binding, protein–protein interactions, and ribosome association were found in patients with human neurodevelopmental disorders [11–14]. In fact, Gemin5 modulates distinct aspects of RNA-dependent processes, executing its functions through the formation of multi-component RNP complexes [19, 46]. An important part of Gemin5 functions require the WD40 domain located at the N-terminal part of the protein [16, 17]. Notwithstanding, other functions rely on the C-terminus of this long multidomain protein, which contains a non-canonical RNA-binding site and shows preference for transcripts comprising stem-loop structures [10]. Additionally, Gemin5 interacts with a large number of RBPs that form part of distinct RNP complexes as a function of the cell physiology, thereby explaining the presence of Gemin5 in diverse RNPs [21, 47]. With the exception of Gemin6 and 7, the transcripts encoding members of the SMN complex were undetected within the mRNAs overrepresented in polysomes upon depletion of Gemin5, consistent with previous work [11]. Future studies will determine if a direct interaction between Gemin5 with distinct mRNAs is the driving force behind the formation of translation competent RNP complexes.

Here, we have shown that Gemin5 is responsible for the preferential association to polysomes of distinct mRNA subsets, which contain specific sequences either on the 5’ or the 3’UTR. These mRNAs encode proteins necessary for two different fundamental cellular processes, protein synthesis and nuclear metabolism, which need to be coordinated for cell growth. We previously identified Gemin5 as a ribosome-associated protein (RAP) [21], in agreement with the ability of Gemin5 to co-sediment with polysomes in living cells [14]. The association of various RBPs to ribosomes has been recognized in numerous studies [32, 48]. Accordingly, it was proposed that subpopulations of protein-associated ribosomes may be responsible for selective regulation of gene expression [49–52]. Here, we propose a model to explain the function of Gemin5 in selective translation (Fig. 7). Among the mRNA targets of Gemin5 only a fraction is differentially associated with polysomes in growing cells. The results obtained in our study led us to hypothesize that a Gemin5-ribosome subpopulation from the ribosome pool is associated with mRNA subsets harboring unique cis-acting elements such as the TOP motif on the 5’end or hSL hairpin at the 3’end. The ability to associate mRNA subsets has previously been observed for ribosomes bound to specific RBPs [52, 53]. Notably, clinical variants of Gemin5 showed decreased levels of Gemin5-ribosome association [14]. Thus, our findings uncover a specialized function for Gemin5 in the association of mRNAs to polysomes, and highlight the impact of ribosome-associated proteins in the pathogenesis linked to altered levels of Gemin5.

## Materials and methods

### Polysomal fractionation

Polysome profiles were prepared from four independent biological samples of control cells and siRNAG5 cells as described [21]. Briefly, HEK293 cells (~ 2 × 10^7^ cells per gradient) were incubated with 0.1 mg/ml cycloheximide (Merck) for 10 min on ice to block ribosomes in the elongation step. Then, cells were washed twice with 0.1 mg/ml cycloheximide in cold PBS, lysed in buffer A (15 mM Tris–HCl pH 7.4, 80 mM KCl, 5 mM MgCl_2_, 0.1 mg/ml cycloheximide), supplemented with 1% (*v*/*v*) Triton X-100, 40 U/ml RNase OUT (Thermo Fisher Scientific), and protease inhibitors (Complete mini, Roche), and centrifuged 10 min at 14,000*g*, 4 °C. The clear supernatants were loaded into a linear 10–50% (*w*/*v*) sucrose gradient in buffer A, centrifuged at 39,000 rpm SW40 Ti rotor 2 h 15 min at 4 °C. Sucrose gradients were fractionated using a gradient fractionation system (ISCO UA-5 UV monitor) measuring the absorbance at A260 to record the polysome profile. Twelve fractions (1 ml) were collected from each gradient.

### RNA isolation

Total RNA from cytoplasmic lysates and polysomal fractions (8–12) was extracted using phenol–chloroform, isopropanol precipitated, and resuspended in 100 µl RNase-free H_2_O. RNA samples were cleaned with the RNeasy kit (Qiagen). RNA quality and concentration were determined using Agilent 2100 Bioanalyzer (Agilent Technologies).

### cDNA libraries preparation and RNA-Seq analysis

The cDNA libraries were prepared with 1 µg of RNA using the Illumina TruSeq Stranded mRNA (Macrogen). RNA sequencing was carried out in an Illumina NovaSeq 6000 platform. The sequencing coverage was 30 M reads per sample and the read length was 100 bp PE.

The quality analysis of the raw sequences performed with FastQC software (version 0.11.7) shows that 96.5% of the sequences have a quality score over 30 and there is negligible percentage of undetermined bases. Sequences were aligned against the human reference genome GRCh38 downloaded from Ensembl release 91 using STAR aligner (version 2.5.3a) [54] with default parameters. Alignment binary files (BAM) including counts were indexed using SAMtools (version 1.7) [55], and the reads were quantified with QoRTs [56]. After grouping of samples (four biological replicates) according to their respective experimental condition, differential gene expressions of siRNAG5 versus Ctrl groups were statistically determined using the R library DESeq2 (version 1.18.1) [57]. Principal components for the individual normalized values were calculated using Singular Value Decomposition (SVD) method of the pcaMethods R package to explore associations between variables [58].

### RTqPCR validation

cDNA synthesis was performed using equal amounts of RNA (1 µg) with SuperScript III (Thermo Fisher Scientific) and hexanucleotide mix (Merck). Quantitative polymerase chain reaction (qPCR) was carried out using three independent biological samples in GoTaq qPCR Master Mix (Promega) on ABI PRISM 7900HT Fast Realtime PCR system (Applied Biosystems). The PCR cycle conditions were 95 °C for 2 min, followed by 40 cycles of 95 °C for 3 s, and 60 °C for 30 s. Cq values were normalized against the mRNA of MYO5A, a constitutively expressed transcript. The primers used for each reaction are shown in Table S6. The comparative cycle threshold method [59] was used to quantify the results.

### Software used for data analysis

Venn diagrams were created using VENNY program available online https://bioinfogp.cnb.csic.es/tools/venny/index2.0.2.html).

Gene Ontology analysis were performed with DAVID V6.8 [36] to identify the biological processes of G5-Enhanced, G5-Repressed, and Gemin5 targets mRNA groups. The cutoff criteria *P* value < 10^–3^ and gene count ≥ 3 was applied.

The Biological Networks Gene Ontology application (BiNGO) was used to analyze the Biological processes of Gemin5-Enhanced targets mRNA group, determining the statistical significance of overrepresented proteins encoded by the mRNAs relative to a complete human proteome [60]. The results were visualized on the Cytoscape platform [61]. The resulting nodes were classified according to a hypergeometric test in the default mode, FDR < 0.05. *P* values of the nodes were computed to find the statistical significance of the network.

STRING software was used to depict the physical and functional interactions among the selected factors. This analysis was carried out using default parameters with a minimum required interaction score of 0.4 [38].

### Constructs

The plasmid pcDNA3Xpress-Gemin5 was previously described [21]. The construct pL32-WT-LUC was generated by inserting the luciferase coding region from pIRES [62] into pL32(1–34)-GH plasmid, kindly provided by Dr. O. Meyuhas [41]. For this, pIRES and pL32(1–34)-GH were digested with SalI and EcoRI, respectively, blunt ended using T4 DNA polymerase, digested again with BamHI, and ligated. The construct pL32-CM6-LUC was generated by QuickChange mutagenesis (Agilent Technologies) using specific primers (Table S6). The constructs Luc-SL and Luc-polyA were prepared ligating and inserting the pair of primers Fw-hSL and Rv-hSL, and Fw-PolyA and Rv-PolyA into pCAP-luc [62] previously linearized with SalI. Similarly, the construct pGEM3-hSL and pGEM3-PolyA were generated ligating the pair of primers Fw-hSL and Rv-hSL and Fw-PolyA and Rv-PolyA, respectively, and then inserted into pGEM3 digested with SalI. All constructs were confirmed by DNA sequencing (Macrogen).

### Gemin5 siRNA interference and expression

siRNAs targeting Gemin5 (CCUUAAUCAAGAAGAGAAAUU) and a control siRNA (AUGUAUUGGCCUGUAUUAGUU) were purchased from Horizon. HEK293 cells grown to 70% confluent were treated with 100 nM siRNA using Lipofectamine 2000 (Thermo Fisher Scientific), 24 h prior to transfection of plasmid Luc-SL linearized with XhoI, or with the plasmids pL32-WT-Luc and pL32-CM6-Luc.

HEK293 cells grown to 70% confluent were cotransfected with the plasmids pL32-WT-Luc or pL32-CM6-Luc, and pcDNA3Xpress-Gemin5 or the empty vector side by side using Lipofectamine LTX (Thermo Fisher Scientific). Likewise, cells were transfected with the plasmid Luc-SL, linearized with XhoI.

### Luciferase activity

Cell lysates were prepared 24 h post-transfection in lysis buffer C (50 mM Tris–HCl pH 7.8, 100 mM NaCl, 0.5% IGEPAL). The concentration of total protein in the lysate was determined by Bradford assay. Equal amounts of protein were loaded in SDS-PAGE and processed for western blotting.

Luciferase activity (RLU) was determined using Berthold Sirius Single Tube Luminometer, and then normalized to the amount of protein in the lysate (RLU/µg). Each experiment was repeated independently at least three times. Values represent the mean ± SEM (standard error of the mean).

### Subcellular fractionation

HEK293 confluent cell monolayers were washed with ice cold PBS, and lysed in buffer 1 (15 mM Tris–HCl pH 7.4, 80 mM KCl, 5 mM MgCl_2_, 1% Triton-X-100, Protease inhibitors (Complete mini, Roche)). Cell debris was discarded by spinning at 14,000*g* 10 min 4 °C. The supernatant (S30 fraction) was centrifuged at 95,000 rpm during 1.5 h using the TLA100.2 rotor, yielding the S100 fraction (supernatant), and the native ribosomes (pellet). Native ribosomes were resuspended in 100 µl of buffer 1. The total protein content in S30 fractions was measured by the Bradford assay; the ribosome concentration was determined as 14 units *A*_260_ = 1 mg/ml.

### Whole cell extracts

Cells were washed twice with ice-cold PBS and harvested in PBS. After spin the cells at 4 °C for 5 min at 800 g, the pellet was incubated with shaking at 4 °C for at least 30 min with 1 V of 50 mM Tris–HCl pH 7.9, 8 M Urea, 1% Chaps. Finally, the supernatant (WCE) was collected after centrifugation 10 min at 14,000*g*, 4 °C.

### Immunodetection

Equal amounts of total protein were resolved on SDS–polyacrylamide gels and transferred to a PVDF membrane (0.2 μm pore, Bio-Rad) using a semi-dry electrotransfer (Bio-Rad). Gemin5 was immunodetected using anti-Gemin5 (Novus) antibody or anti-Xpress (Thermo Fisher Scientific). Commercial antibodies were used to detect RPL3 and RACK1 (Santa Cruz). P1 and P2 ribosomal proteins were detected with the monoclonal antibody 3BH5 [63]. Anti-H3A (abcam) and Histone H2A (Cell Signaling) were a kind gift from Dr. C. Gutierrez and Dr. E. Lecona. Immunodetection of tubulin (Merck) was used as loading control. The appropriate secondary HRP-conjugated antibodies (Thermo Fisher Scientific) were used according to the instructions of the manufacturer.

### RNA immunoprecipitation

HEK293 cells were washed with ice-cold PBS prior to UV irradiation (150 mJ/cm^2^, 254 nm) and lysed at 4 °C in lysis buffer (25 mM Tris–HCl pH 7.5, 150 mM NaCl, 5 mM EDTA, 10 mM β-Glycerol phosphate, 0.2 U/µl of RNaseOUT (Thermo Fisher Scientific), 1% Triton-X-100, and protease inhibitors (Complete mini, Roche)). Cells were broken by passing them through a 22G-needle, and centrifuged for 10 min at 4 °C. The cleared supernatant was then incubated with 20 μl Dynabeads™ Protein A (Thermo Fisher Scientific) magnetic beads previously conjugated with 1 μg anti-Gemin5 antibody or rabbit IgG antibody (Merck) for 3 h at 4 °C with shaking. After three washing steps, beads and total cell lysate were incubated with 200 μl Proteinase K buffer (50 mM Tris–HCl pH 7.5, 150 mM NaCl, 0.5% SDS, 5 mM MgCl_2_, 0.2 mg/ml proteinase K) at 65 °C 2 h with gentle rocking. RNAs were then extracted and resuspended in 20 μl RNase-free H_2_O. Reverse transcriptase was carried out to synthesize cDNA from equal amounts of the purified RNA samples. qPCR was performed using iTaq Universal SYBR qPCR Master Mix (Bio-Rad) on an CFX-384 Fast Realtime PCR system (Bio-Rad). The pairs of primers targeting specific ribosomal and histone mRNAs used for qPCR are listed in Table S6.

### Protein affinity purification

Confluent HEK293 cell monolayers were co-transfected with pcDNA3Xpress-Gemin5, and pL32-WT-Luc or pL32-CM6-Luc. Monolayers were washed 24 h post-transfection with ice-cold PBS prior to UV irradiation (400 mJ/cm^2^ at 254 nm). Ni–NTA Agarose (Qiagen) was used for purification of His-tagged proteins by gravity-flow chromatography according to the manufacturer's instructions. After washing, purified samples were treated with proteinase K and RNAs were extracted as described above.

### RNA synthesis and in vitro translation assays

pLuc-hSL and pLuc-polyA constructs containing the consensus histone stem-loop or a poly(A) tail downstream of the luciferase open reading frame were linearized with XhoI and NdeI, respectively, immediately downstream of the stem-loop or the poly(A) tail. Transcription was performed for 2 h at 37 °C using 10–50 U of T7 RNA polymerase in the presence of 1–3 µg of linearized DNA template, 40 mM Tris–HCl, 50 mM DTT, 0.5 mM rNTPs. RNA was extracted with phenol–chloroform, ethanol precipitated and resuspended in TE (10 mM Tris–HCl pH 8, 1 mM EDTA).

Luc-SL and Luc-p(A) RNAs were translated in 70% rabbit reticulocyte lysate (RRL) (Promega) supplemented with increasing amounts of human Gemin5 protein (0–400 ng) (Origene). Luciferase activity (RLU) was analyzed to monitor translation efficiency.

### RNA electrophoretic mobility shift assay

hSL and polyA RNAs uniformly labelled probes were prepared using *α*^32^P-CTP (500 Ci/mmol), T7 RNA polymerase (10 U), and linearized pGEM3-hSL or pGEM3-PolyA plasmids (1 μg) with XhoI and NdeI, respectively. hSL and PolyA RNAs were purified through MicroSpin G-25 columns (GE Healthcare), ethanol precipitated, and resuspended in TE to a final concentration of 0.04 pmol/µl. RNA integrity and mobility as a single band was examined in 6% acrylamide 7 M urea denaturing gel electrophoresis [44].

Prior to RNA-binding, in vitro synthesized hSL and polyA RNAs were heated at 95 °C for 2 min, snap cooling on ice for 2 min, and subsequently incubated in folding mix (100 mM HEPES pH 8.0, 100 mM NaCl, 5.25 mM MgCl_2_) for 20 min at 37 °C. Then, RNA-binding reactions were carried out in 10 µl of RNA-binding buffer (40 mM Tris–HCl pH 7.5, 250 mM NaCl, 0.1% (*w*/*v*) βME) for 15 min at room temperature. Purified Gemin5 protein (200, 400 ng- µM) (Origene) was incubated with ^32^P-labeled RNA (about 2 nM). Electrophoresis was performed in non-denaturing 6.0% (29:1) polyacrylamide gels. The gels were run at 4 °C in TBE buffer (90 mM Tris–HCl pH 8.4, 64.6 mM boric acid, 2.5 mM EDTA) at 100 V for 1 h. The ^32^P-labelled RNA and retarded complexes were detected by autoradiography of dried gels.

### Statistical analyses

Statistical analyses for RTqPCR and luciferase activity were performed as follows. Each experiment was repeated independently at least three times. Values represent the estimated mean ± SEM. Difference in distribution between two samples was analyzed by paired two-tailed Student’s *t* test, and were considered significant when *P* < 0.05. The resulting *P* values were graphically illustrated in figures with asterisks as described in figure legends.

## Availablity of data and material

The accession code for the FASTQ files and raw gene counts reported in this paper are deposited in GEO under the accession number GSE200388. All data generated or analyzed during this study are included in the manuscript and supporting files.

### Supplementary Information

Below is the link to the electronic supplementary material.

Supplementary file1 (PDF 790 KB)

Supplementary file2 (XLSX 140 KB)
