# Supplementary material for: Gemin5-dependent RNA association with polysomes enables selective translation of ribosomal and histone mRNAs
Source: Cell Mol Life Sci. 2022 Aug 20;79(9):490. doi: 10.1007/s00018-022-04519-4 (PMC9392717; doi:10.1007/s00018-022-04519-4)
Supplement: Supplementary file 1 — Supplementary file1 (PDF 790 KB) [file 18_2022_4519_MOESM1_ESM.pdf]

Gemin5-dependent RNA association with polysomes enables selective translation of ribosomal and histone mRNAs

Azman Embarc-Buh[1,#], Rosario Francisco-Velilla[1,#], Juan Antonio Garcia-Martin[2], Salvador Abellan[1], Jorge Ramajo[1], and Encarnacion Martinez-Salas[1,*]

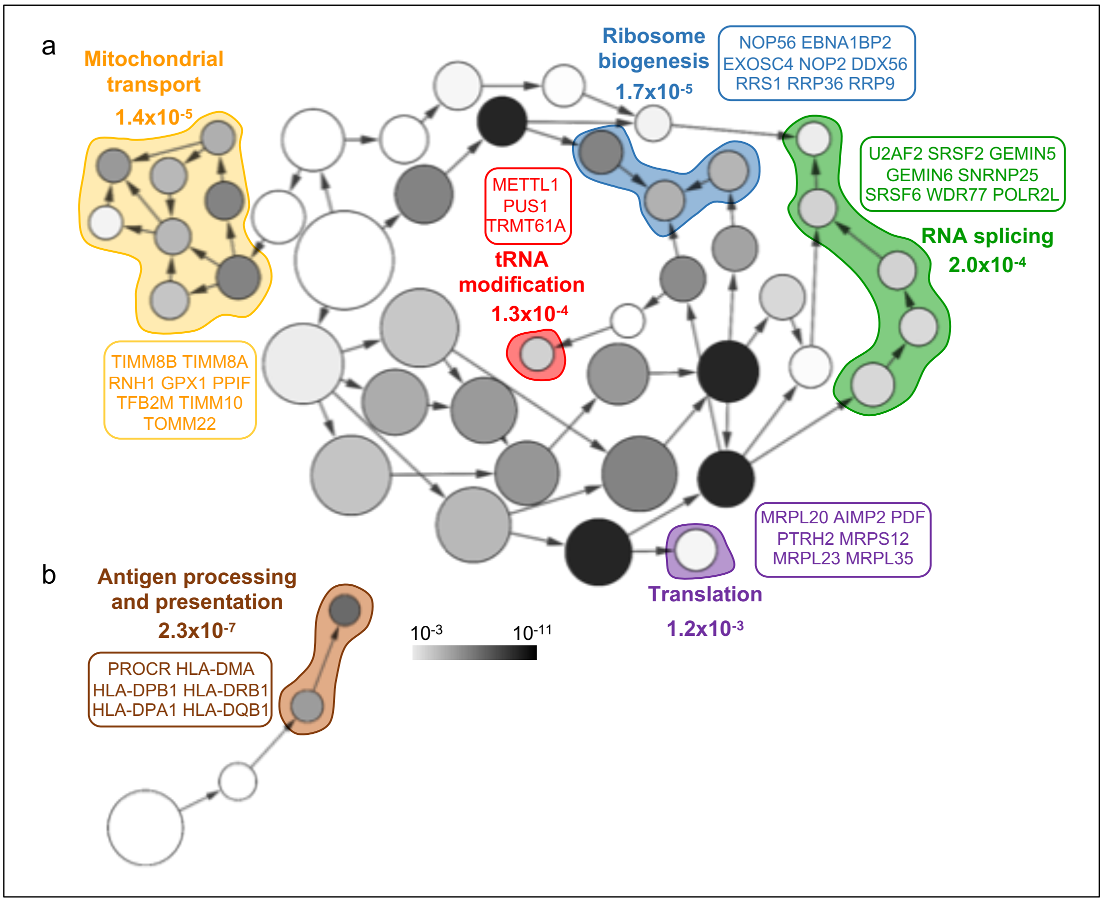

Fig. S1. Functional networks obtained with BiNGO (Cytoscape platform) of proteins encoded by mRNAs down-represented (a) or over-represented in Input (b). The *P*-values of the networks relative to a complete human proteome, and the proteins that compose the nodes included in each network are indicated.

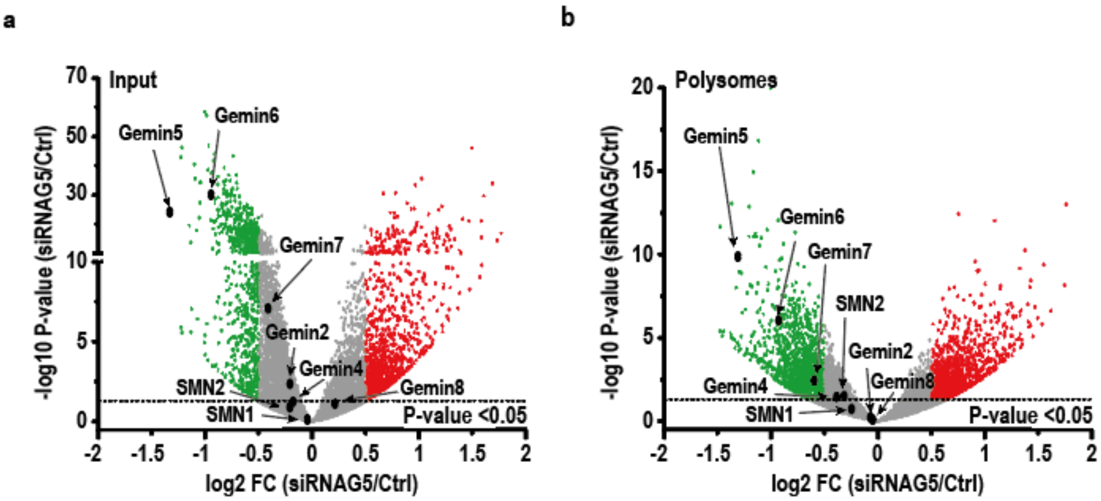

Fig. S2. Volcano plots showing the log2 FC (X) versus -log10 *P* value (Y) of the mRNAs encoding proteins of the SMN complex detected in the RNA-Seq analysis from the four replicas for the Input (a), and the Polysome (b), using as cut off -0.5 > log2 FC > 0.5 and *P* < 0.05.

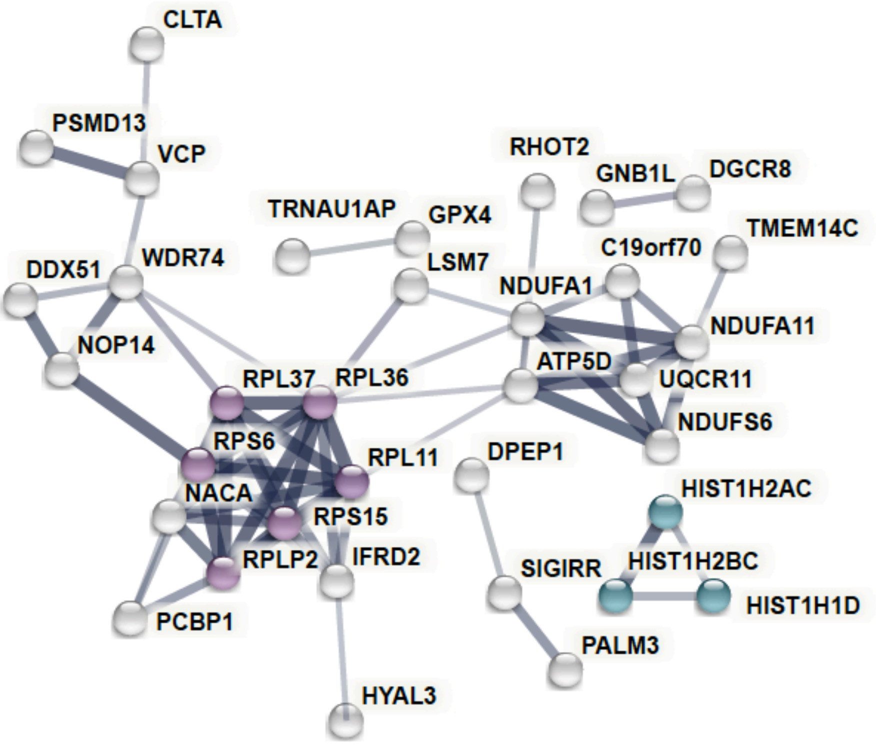

Fig. S3. STRING networks of predicted associations for the proteins encoded by the polysome-bound mRNA targets of Gemin5.

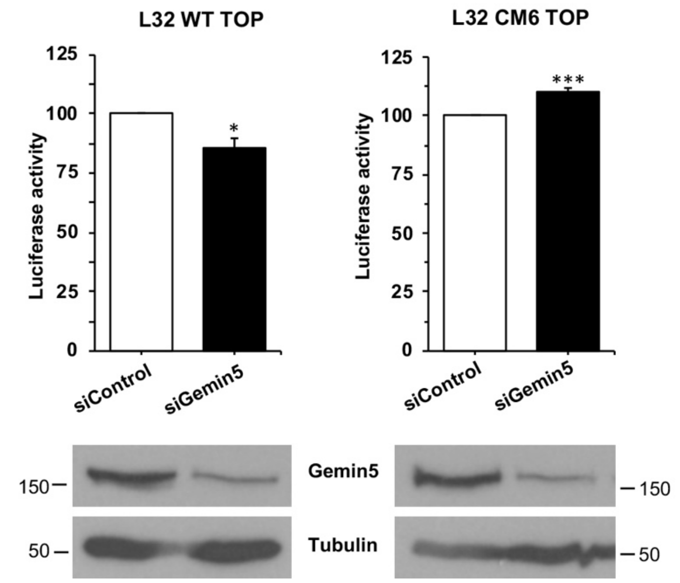

Fig. S4. Effect of the Gemin5 silencing on the translation of the L32 WT TOP or the mutant L32 CM6 TOP mRNAs. Gemin5 depleted and control cells were transfected with the indicated plasmids to monitor luciferase activity. Gemin5 silencing was monitored by western blot using anti-Gemin5 antibody. Tubulin was used as loading control.

Table S1. TOP mRNAs

Gene ID	Gene name	L2FC (siRNAG5/Ctrl)	-Log10P-value (siRNAG5/Ctrl)
ENSG00000165502	RPL36AL	-0.97	6.82
ENSG00000182899	RPL35A	-0.94	4.32
ENSG00000122026	RPL21	-0.93	4.19
ENSG00000136942	RPL35	-0.93	4.27
ENSG00000170889	RPS9	-0.92	4.77
ENSG00000171858	RPS21	-0.88	3.00
ENSG00000198918	RPL39	-0.87	3.36
ENSG00000083845	RPS5	-0.87	3.88
ENSG00000172809	RPL38	-0.86	3.78
ENSG00000143947	RPS27A	-0.85	3.65
ENSG00000130255	RPL36	-0.81	3.26
ENSG00000108107	RPL28	-0.81	3.18
ENSG00000177600	RPLP2	-0.81	2.98
ENSG00000115268	RPS15	-0.81	3.48
ENSG00000241343	RPL36A	-0.80	2.11
ENSG00000166441	RPL27A	-0.80	2.84
ENSG00000105193	RPS16	-0.80	3.10
ENSG00000104529	EEF1D	-0.79	5.38
ENSG00000213741	RPS29	-0.78	2.32
ENSG00000233927	RPS28	-0.77	2.36
ENSG00000071082	RPL31	-0.77	3.47
ENSG00000008988	RPS20	-0.77	2.82
ENSG00000221983	UBA52	-0.76	3.23
ENSG00000144713	RPL32	-0.76	2.95
ENSG00000118181	RPS25	-0.75	2.91
ENSG00000149806	FAU	-0.75	3.26
ENSG00000231500	RPS18	-0.75	2.82
ENSG00000186468	RPS23	-0.75	2.82
ENSG00000147403	RPL10	-0.74	3.12
ENSG00000164587	RPS14	-0.74	2.79
ENSG00000147604	RPL7	-0.74	3.22
ENSG00000105640	RPL18A	-0.74	2.67
ENSG00000110700	RPS13	-0.74	2.92
ENSG00000156482	RPL30	-0.73	2.86
ENSG00000177954	RPS27	-0.73	2.14
ENSG00000125691	RPL23	-0.72	3.06
ENSG00000162244	RPL29	-0.72	3.02
ENSG00000105372	RPS19	-0.71	2.45
ENSG00000131469	RPL27	-0.71	2.69
ENSG00000137154	RPS6	-0.71	2.70
ENSG00000116251	RPL22	-0.70	2.99
ENSG00000197756	RPL37A	-0.70	2.22
ENSG00000229117	RPL41	-0.70	2.09

ENSG00000171863	RPS7	-0.70	2.97
ENSG00000197958	RPL12	-0.68	2.29
ENSG00000112306	RPS12	-0.68	2.02
ENSG00000182774	RPS17	-0.68	2.18
ENSG00000145592	RPL37	-0.67	2.24
ENSG00000198242	RPL23A	-0.67	2.52
ENSG00000148303	RPL7A	-0.65	3.03
ENSG00000109475	RPL34	-0.65	2.25
ENSG00000108298	RPL19	-0.63	2.64
ENSG00000142676	RPL11	-0.63	2.44
ENSG00000161016	RPL8	-0.62	3.04
ENSG00000198755	RPL10A	-0.61	2.31
ENSG00000114391	RPL24	-0.60	2.41
ENSG00000089157	RPLP0	-0.60	1.99
ENSG00000188846	RPL14	-0.60	2.50
ENSG00000114942	EEF1B2	-0.59	2.54
ENSG00000142541	RPL13A	-0.59	1.70
ENSG00000142534	RPS11	-0.57	1.92
ENSG00000138326	RPS24	-0.57	2.16
ENSG00000122406	RPL5	-0.50	2.27

Table S2. Histone mRNAs

Gene ID	Gene name	L2FC (siRNAG5/Ctrl)	-Log10P-value (siRNAG5/Ctrl)
ENSG00000197238	H4C11	-1.27	6.52
ENSG00000278463	H2AC4	-1.12	3.53
ENSG00000274267	H3C2	-1.04	3.99
ENSG00000180596	H2BC4	-1.01	3.60
ENSG00000181218	H2AW	-0.95	4.78
ENSG00000275714	H3C1	-0.93	2.75
ENSG00000183598	H3C13	-0.93	2.98
ENSG00000184270	H2AC21	-0.87	2.25
ENSG00000278828	H3C10	-0.80	2.86
ENSG00000274641	H2BC17	-0.79	2.21
ENSG00000196747	H2AC13	-0.78	2.90
ENSG00000124575	H1-3	-0.72	2.00
ENSG00000275221	H2AC15	-0.68	1.43
ENSG00000124635	H2BC11	-0.65	2.39
ENSG00000196787	H2AC11	-0.64	1.70
ENSG00000274750	H3C6	-0.62	1.50
ENSG00000277157	H4C4	-0.62	1.42
ENSG00000246705	H2AJ	-0.61	2.73
ENSG00000180573	H2AC6	-0.50	1.34

Table S3. Enhanced mRNAs

Gene ID	Gene name	L2FC (siRNAG5/Ctrl)	-Log10P-value (siRNAG5/Ctrl)
ENSG00000146674	IGFBP3	-1.48	5.40
ENSG00000011465	DCN	-1.44	5.40
ENSG00000260924	LINC01311	-1.38	5.27
ENSG00000163581	SLC2A2	-1.37	5.04
ENSG00000113600	C9	-1.37	5.01
ENSG00000146678	IGFBP1	-1.35	4.93
ENSG00000143546	S100A8	-1.34	4.87
ENSG00000263934	SNORD3A	-1.34	4.41
ENSG00000180210	F2	-1.32	4.74
ENSG00000134389	CFHR5	-1.30	4.55
ENSG00000280273	AF131216.4	-1.30	4.22
ENSG00000002933	TMEM176A	-1.29	4.34
ENSG00000197238	HIST1H4J	-1.27	6.52
ENSG00000129988	LBP	-1.25	3.99
ENSG00000152583	SPARCL1	-1.23	4.10
ENSG00000079557	AFM	-1.23	4.30
ENSG00000187758	ADH1A	-1.22	4.33
ENSG00000148346	LCN2	-1.20	4.04
ENSG00000110436	SLC1A2	-1.18	3.91
ENSG00000189056	RELN	-1.17	4.02
ENSG00000260260	SNHG19	-1.15	4.30
ENSG00000021852	C8B	-1.15	3.91
ENSG00000261701	HPR	-1.15	3.91
ENSG00000167711	SERPINF2	-1.15	3.92
ENSG00000198610	AKR1C4	-1.14	3.89
ENSG00000140093	SERPINA10	-1.14	3.88
ENSG00000139220	PPFIA2	-1.12	3.63
ENSG00000186529	CYP4F3	-1.12	3.64
ENSG00000278463	HIST1H2AB	-1.12	3.53
ENSG00000273619	AL121832.2	-1.11	5.97
ENSG00000164692	COL1A2	-1.11	3.73
ENSG00000101076	HNF4A	-1.11	3.74
ENSG00000130997	POLN	-1.09	3.78
ENSG00000106538	RARRES2	-1.07	3.60
ENSG00000247092	SNHG10	-1.07	4.99
ENSG00000132840	BHMT2	-1.06	3.56
ENSG00000274267	HIST1H3B	-1.04	3.99
ENSG00000132855	ANGPTL3	-1.04	3.47
ENSG00000174721	FGFBP3	-1.04	4.24
ENSG00000242125	SNHG3	-1.03	7.86
ENSG00000106327	TFR2	-1.03	3.41
ENSG00000180596	HIST1H2BC	-1.01	3.60
ENSG00000123843	C4BPB	-1.01	3.32

ENSG00000165502	RPL36AL	-0.97	6.82
ENSG00000181218	HIST3H2A	-0.95	4.78
ENSG00000102030	NAA10	-0.95	5.42
ENSG00000182899	RPL35A	-0.94	4.32
ENSG00000122026	RPL21	-0.93	4.19
ENSG00000136942	RPL35	-0.93	4.27
ENSG00000227063	RPL41P1	-0.92	3.37
ENSG00000148291	SURF2	-0.92	5.11
ENSG00000235174	RPL39P3	-0.92	3.55
ENSG00000170889	RPS9	-0.92	4.77
ENSG00000269893	SNHG8	-0.92	3.64
ENSG00000102103	PQBP1	-0.90	7.58
ENSG00000243199	AC115223.1	-0.89	4.02
ENSG00000099849	RASSF7	-0.88	6.69
ENSG00000155016	CYP2U1	-0.88	4.28
ENSG00000198918	RPL39	-0.87	3.36
ENSG00000235065	RPL24P2	-0.87	3.61
ENSG00000083845	RPS5	-0.87	3.88
ENSG00000214309	MBLAC1	-0.87	4.50
ENSG00000172809	RPL38	-0.86	3.78
ENSG00000147996	CBWD5	-0.85	4.38
ENSG00000143947	RPS27A	-0.85	3.65
ENSG00000261526	AC012615.1	-0.84	3.54
ENSG00000132330	SCLY	-0.84	4.73
ENSG00000104356	POP1	-0.84	3.55
ENSG00000135763	URB2	-0.82	4.01
ENSG00000164346	NSA2	-0.81	6.54
ENSG00000169857	AVEN	-0.81	5.71
ENSG00000115268	RPS15	-0.81	3.48
ENSG00000173638	SLC19A1	-0.81	9.77
ENSG00000089009	RPL6	-0.80	4.14

Table S4. Repressed mRNAs

Gene ID	Gene name	L2FC (siRNAG5/Ctrl)	-Log10P-value (siRNAG5/Ctrl)
ENSG00000188185	LINC00265	1.32786382	6.30619717
ENSG00000261716	AC239868.2	1.2556284	3.96131711
ENSG00000182040	USH1G	1.21971734	4.05170648
ENSG00000155761	SPAG17	1.1914491	3.67261991
ENSG00000113763	UNC5A	1.18917413	3.99038716
ENSG00000004776	HSPB6	1.17125973	3.58458134
ENSG00000129682	FGF13	1.14176941	3.95447743
ENSG00000187135	VSTM2B	1.13690897	3.73772593
ENSG00000188177	ZC3H6	1.12351031	6.28789884
ENSG00000248243	LINC02014	1.10352093	3.49920859

ENSG00000102053	ZC3H12B	1.10351455	4.19259081
ENSG00000080298	RFX3	1.09756251	4.66372381
ENSG00000048392	RRM2B	1.09104386	12.0403601
ENSG00000280123	AC023632.6	1.08333991	4.96018425
ENSG00000232907	DLGAP4-AS1	1.06145311	3.76005756
ENSG00000188523	CFAP77	1.04697063	3.56222485
ENSG00000111846	GCNT2	1.03750733	5.17859007
ENSG00000141639	MAPK4	1.03632509	3.80953587
ENSG00000168135	KCNJ4	1.03053001	3.58732842
ENSG00000261115	TMEM178B	1.02149568	4.70604879
ENSG00000064692	SNCAIP	1.0131938	3.44911472
ENSG00000102531	FNDC3A	1.0043344	6.38059171
ENSG00000101752	MIB1	1.0020629	4.68736518
ENSG00000173273	TNKS	1.00090198	5.97438139
ENSG00000139793	MBNL2	0.99662544	4.96607358
ENSG00000150625	GPM6A	0.98906446	3.36011313
ENSG00000162636	FAM102B	0.95232831	5.72967384
ENSG00000112182	BACH2	0.9496011	3.66481271
ENSG00000213626	LBH	0.93732215	5.73794877
ENSG00000237517	DGCR5	0.92864885	3.92329388
ENSG00000112624	BICRAL	0.92301907	3.38342871
ENSG00000144369	FAM171B	0.91752981	5.42983633
ENSG00000174738	NR1D2	0.91182132	9.57748376
ENSG00000278709	NKILA	0.90945171	3.62797533
ENSG00000111252	SH2B3	0.90482979	4.90617288
ENSG00000155304	HSPA13	0.89407348	3.9371653
ENSG00000198961	PJA2	0.8911033	3.76909704
ENSG00000169891	REPS2	0.88003415	4.86369978
ENSG00000083290	ULK2	0.87620184	4.35108548
ENSG00000106100	NOD1	0.87036125	3.44733394
ENSG00000141682	PMAIP1	0.86355095	7.56786794
ENSG00000168214	RBPJ	0.86307945	6.08748049
ENSG00000169499	PLEKHA2	0.85288314	3.50222402
ENSG00000160712	IL6R	0.85243774	3.66697263
ENSG00000227372	TP73-AS1	0.85151536	4.15480712
ENSG00000236609	ZNF853	0.84704587	3.58975406
ENSG00000120693	SMAD9	0.84698194	5.59290324
ENSG00000120071	KANSL1	0.84381574	5.85455548
ENSG00000004777	ARHGAP33	0.84164582	3.31851997
ENSG00000168137	SETD5	0.84098248	5.35618297
ENSG00000104490	NCALD	0.83878495	3.85801494
ENSG00000158106	RHPN1	0.8346893	3.765706
ENSG00000175093	SPSB4	0.82219461	5.37692733
ENSG00000248092	NNT-AS1	0.80356354	5.02224674

Table S5. TOP and histone Gemin5 targets

Gene ID	Gene name	Gene ID	Gene name
ENSG00000145592	RPL37	ENSG00000180573	H2AC6
ENSG00000177600	RPLP2	ENSG00000180596	H2BC4
ENSG00000137154	RPS6	ENSG00000197409	H3C4
ENSG00000124614	RPS10	ENSG00000158406	H4C8
ENSG00000115268	RPS15	ENSG00000187837	H1-2
ENSG00000134419	RPS15A	ENSG00000124575	H1-3
ENSG00000130255	RPL36	ENSG00000168298	H1-4
ENSG00000167658	EEF2	ENSG00000196866	H2AC7
ENSG00000163682	RPL9	ENSG00000158373	H2BC5
ENSG00000142676	RPL11	ENSG00000203811	H3C14
		ENSG00000197697	H2BC6

Table S6. Primers

Gene	Forward (5'-3')	Reverse (5'-3')
**Gemin5**	AAGGTGAGCGAATGCTGAGT	GGATCATTTCTGCCAAGGTC
**DDX39**	TGAGGACTCGGACACCTACC	TTTTGGCATCATTCTCGTCA
**RRS1**	CAGCCGAGAAAAAGAACCAG	CCTCCTCCCTCATCTGCTTA
**RBM3**	ATTGGTTCTGGCAAGTTTGG	TGGGAAATTGCTTCCTAACC
**TP53INP1**	CTGTTTAGGGTGGAGAAAGAGGT	TGCCTGCCTTTCAAGAGTAACT
**RRM2B**	GAGGAAGGGCTTATGGACTGAG	TGCTGGTACACTATTCAGGCTG
**RHEBL1**	GTCGCTGCCATCTCATGTGA	TACCCGTGAAGTCCTGAGGA
**LINC00324**	TCCAGGAAAACAGCGATCGA	AGGTTCATGTGGGGTGGGT
**MYO5A**	TCGCCATATCTGTCTCATCATC	AGGGAAGAACGTCTTGCACA
**pL32-CM6**	GGCAGCGCCGAGGTTCATCGAACAGGCGGCG GCGGCGC	GCGCCGCCGCCGCCTGTTCGATGAACCTCGG CGCTGCC
**hSL**	TCGACCAAAAGGCTCTTTTCAGAGCCACCCAC TCGAGG	TCGACCTCGAGTGGGTGGCTCTGAAAAGAGC CTTTTGG
**PolyA**	TCGACAAAAAAAAAAAAAAAAAAAAAAAAA AAAAAAAAAAAAAAAAAAAAAAACATATGG	TCGACCATATGTTTTTTTTTTTTTTTTTTTTTT TTTTTTTTTTTTTTTTTTTTTTTTTG
**RPL32**	TGCCGAGATCGCTCACAATG	GCATTGGGGTTGGTGACTCT
**RPL3**	AATGGCAGGATGAGGATGGC	CATCTGGGTGTGGGCAATGA
**RPLP1**	CCCTCATTCTGCACGACGAT	CCAGGCCAAAAAGGCTCAAC
**RACK1**	TGCTTCTGGAGGCAAGGATG	AAGCACAGGGCGTTGATGAT
**RPL35**	GAAACAGCTGGACGACCTGA	TCCGGACGACTCGGATCTTA
**RPS9**	CGCTTGATGAGAAGGACCCA	CCAGCTTCATCTTGCCCTCA
**RPL21**	CTTTGGCCACATATATGCGAATC	GGTAACACTTGTGGGGCATTC
**H2AC4**	AAACTCTTGGGGCGTGTGAC	CCTTGGCCTTATGATGGCTCT
**H3C1**	TAGTGTTGGGTGTTCCGCTG	GCCTTAGTGGCCAACTGTTT
**H1**	CATCCGGACCCCCAGTATCT	CTTTCTTAAGCGCGGCCAGA
**H2AC6**	GCTTAGGCCGCTGGTTTTG	AAGAGCGGGATTTCGCTTTGG
